# Tracking Chaperone-Mediated Autophagy Flux with a pH-Resistant Fluorescent Reporter

**DOI:** 10.3390/ijms26010017

**Published:** 2024-12-24

**Authors:** Ruotong Qi, Xingyi Chen, Zihan Li, Zheng Wang, Zhuohui Xiao, Xinyue Li, Yuanyuan Han, Hongfei Zheng, Yanjun Wu, Yi Xu

**Affiliations:** Shanghai Key Laboratory of Metabolic Remodeling and Health, Institute of Metabolism and Integrative Biology, Fudan University, Shanghai 200438, China; 22210880012@m.fudan.edu.cn (R.Q.); 21110880006@m.fudan.edu.cn (X.C.); 23210880007@m.fudan.edu.cn (Z.L.); 24210880013@m.fudan.edu.cn (Z.W.); 22110880019@m.fudan.edu.cn (Z.X.); 23110880013@m.fudan.edu.cn (X.L.); 23110880005@m.fudan.edu.cn (Y.H.); 24110880026@m.fudan.edu.cn (H.Z.)

**Keywords:** chaperone-mediated autophagy, flux, halo, Gamillus

## Abstract

Chaperone-mediated autophagy (CMA) is a selective autophagic pathway responsible for degrading cytoplasmic proteins within lysosomes. Monitoring CMA flux is essential for understanding its functions and molecular mechanisms but remains technically complex and challenging. In this study, we developed a pH-resistant probe, KFERQ-Gamillus, by screening various green fluorescent proteins. This probe is activated under conditions known to induce CMA, such as serum starvation, and relies on LAMP2A and the KFERQ motif for lysosomal localization and degradation, demonstrating its specificity for the CMA pathway. It enables the detection of CMA activity in living cells through both microscopy and image-based flow cytometry. Additionally, we created a dual-reporter system, KFERQ-Gamillus-Halo, by integrating KFERQ-Gamillus with the Halo-tag system. This probe not only distinguishes between protein synthesis and degradation but also facilitates the detection of intracellular CMA flux via immunoblotting and the rapid assessment of CMA activity using flow cytometry. Together, the KFERQ-Gamillus-Halo probe provides quantitative and time-resolved monitoring for CMA activity and flux in living cells. This tool holds promising potential for high-throughput screening and biomedical research related to CMA.

## 1. Introduction

Autophagy (‘self-eating’) is a conserved cellular process that degrades various molecules and subcellular components through lysosome-mediated pathways, thereby preserving cellular homeostasis [1,2]. The degradation of intracellular proteins and organelles via autophagy occurs in three primary forms: macroautophagy, microautophagy, and chaperone-mediated autophagy (CMA). Each form has unique physiological functions and specific mechanisms for delivering cargo to the lysosome [3]. Autophagy is upregulated in response to nutrient deprivation or external stress, serving as a self-protective mechanism to clear invading pathogens or cellular waste and recycle energy. It plays a critical role in physiological processes such as cellular homeostasis, aging, and immunity. However, the dysregulation of autophagy can lead to metabolic dysfunction and is associated with cancer, diabetes, and neurodegenerative diseases [1].

The autophagic degradation of substrates in the lysosome can be either selective or non-selective. Notably, selective degradation was first described in CMA [4,5,6]. CMA selectively degrades proteins directed to lysosomes by the cytosolic chaperone Hsc70 (heat shock-cognate 70 kDa) [6], which recognizes and binds a specific pentapeptide motif (KFERQ-like) within the substrate protein [7]. At the lysosome, the substrate/Hsc70 complex binds to the cytosolic tail of lysosome-associated membrane protein type 2A (LAMP2A) [5], a rate-limiting protein in CMA and one of three splice variants encoded by the *Lamp2* gene. This binding initiates the oligomerization of LAMP2A into a multimeric complex, facilitating substrate translocation into the lysosome. A lysosomal-resident isoform of Hsc70 assists in translocating the substrate into the lysosomal lumen, where it is subsequently degraded by acidic hydrolases [8]. It is estimated that approximately 30% of cytosolic proteins, including oxidized or damaged ones, are degraded through CMA [9,10]. CMA is present in nearly all experimentally tested mammalian cells, though its activity varies [11]. It increases under conditions such as serum starvation [12], hypoxia [13], oxidative stress [14], differentiation [15], and DNA damage [16]. CMA activity declines with age [17] and in response to metabolic challenges, such as high-lipid diets [18]. CMA has been demonstrated to play a pivotal role in diverse physiopathological processes, including metabolism [19,20], circadian rhythm [21], immune responses [22,23], stem cell homeostasis [15,24], and neurodegenerative diseases [25].

Monitoring autophagic activity is essential for understanding cellular processes and advancing therapeutic development. However, accurately quantifying autophagic flux, particularly in CMA, remains challenging. Unlike macroautophagy, CMA selectively degrades individual substrates, which adds technical complexity to its study. Conventional methods for analyzing CMA activity, such as in vitro lysosome isolation, ^3^H-leucine metabolic radiolabeling, and substrate binding and uptake assays, have provided valuable insights into this selective autophagic pathway [26]. However, these techniques typically require large cell quantities, are disruptive, and are labor-intensive. These limitations hinder the real-time analysis of primary differentiated and non-dividing cultured cells, such as neurons and cardiomyocytes, and prevent the assessment of CMA activity across distinct cell types within a specific tissue or microenvironment. To monitor CMA activity in vitro and in vivo, researchers have developed monomeric fluorescent reporters, KFERQ-PA-mCherry and KFERQ-Dendra, which are photoactivatable (PA) and photoconvertible, respectively. These reporters exploit the mechanism by which the fusion of the KFERQ motif to a non-CMA substrate facilitates its degradation through CMA. Specifically, the reporters were engineered by fusing the first 21 amino acids of ribonuclease A (RNase A), the first protein identified as a CMA substrate and containing a KFERQ motif, to the fluorescent markers PA-mCherry or Dendra [11,27]. However, the widely used in vitro KFERQ-PA-mCherry CMA reporter has several limitations: it requires photoexcitation with 405 nm light, which is time-consuming, prone to photobleaching, and unsuitable for extended observation. It also lacks sensitivity for detecting low CMA activity and does not provide an internal standard for normalization. Additionally, it cannot directly monitor CMA flux in live cells, limiting its application in genome-wide high-throughput screening. Recently, a KFERQ-AMC fluorogenic CMA substrate was synthesized and used to monitor CMA activity in cells and tissues. However, this method cannot measure CMA flux and requires the use of lysosomal inhibitors [28]. Attempts to use KFERQ-EGFP (KFERQ-fused enhanced green fluorescent protein) to measure CMA activity have faced significant challenges. Like other CMA substrates, this reporter must fully unfold before lysosomal translocation, resulting in a loss of fluorescence in the acidic lysosomal environment. Only the small fraction that binds to the lysosomal membrane remains visible, which does not clearly delineate lysosomes compared to cytosolic proteins [11]. Therefore, the development of a pH-resistant green fluorescent protein would significantly expand the toolkit for tracking CMA activity.

Measuring CMA flux is essential but challenging. The GFP-LC3-RFP-LC3ΔG fluorescent probe, initially developed to evaluate macroautophagy flux, has limited time resolution and depends on endogenous ATG4 expression levels [29]. More recently, a pulse-chase Halo-Tag-based LC3B reporter assay was introduced for monitoring macroautophagy flux in mammalian cells. This approach selectively labels extra-lysosomal proteins and enables the detection of autophagic flux by tracking free Halo ligands [30]. Furthermore, CMA activity has been successfully tracked in single neurons using a GAPDH-Halo-tag fusion [31]. However, this probe relies on full-length GAPDH, which may be influenced by protein aggregation and signaling pathways regulating GAPDH expression. Collectively, these studies highlight the potential of the Halo-tag as a tool for monitoring autophagy flux.

In this study, we set out to develop an enhanced fluorescent marker for the real-time tracking of CMA flux in single cells. By screening various KFERQ-fused GFP variants, we successfully identified a pH-resistant KFERQ-Gamillus fusion protein that facilitates the dynamic and precise monitoring of CMA activity. This fusion protein displays a visible punctate pattern, making it suitable for both microscopy and image-based flow cytometry. Additionally, we created a dual-reporter system, KFERQ-Gamillus-Halo, by combining it with a Halo-tag. This system not only mirrors photoactivatable reporters in its ability to distinguish protein synthesis from degradation but also enables the detection of intracellular CMA flux via immunoblotting and rapid CMA activity assessment using flow cytometry. Together, KFERQ-Gamillus and KFERQ-Gamillus-Halo serve as versatile, pH-resistant fluorescent reporters for monitoring CMA activity and flux. These tools hold great potential for high-throughput screening and advancing biomedical research to further elucidate the pathophysiological roles of CMA.

## 2. Results

### 2.1. Screening of Green Fluorescent Proteins for CMA Reporter

**Figure 1:**
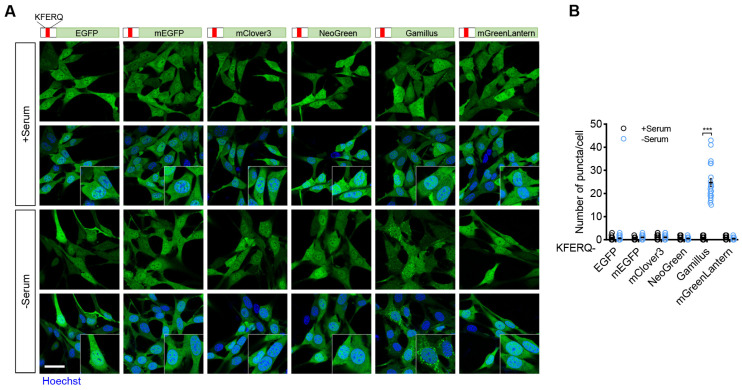
Screening of green fluorescent proteins for CMA reporter. (**A**,**B**) NIH-3T3 cells stably expressing KFERQ-fused fluorescent proteins, as indicated, were cultured in medium with (+Serum) or without (−Serum) serum for 24 h. Shown are representative cell images (**A**) and average number of puncta per cell (**B**) (*n* = 20 for each of three independent experiments). Scale bar, 10 μm. Data are shown as mean ± SD; *** *p* < 0.001; unpaired Student’s *t*-test.

To monitor CMA activity in living cells, a previous study demonstrated that fusing monomeric green fluorescent proteins with the first 21 amino acids of RNase A, which contains a KFERQ-like motif, enables these proteins to act as CMA substrates. Once targeted for lysosomal degradation, these substrates illuminate the compartments involved in CMA activity [27]. However, the green fluorescent puncta generated are quickly quenched in the acidic lysosomal environment. To overcome this limitation, we sought to identify a fluorescent protein capable of tracking CMA activity within the acidic lysosome at the single-cell level. We generated several stable cell lines expressing KFERQ-motif-fused green fluorescent proteins with varying aggregation states, fluorescence intensities, and levels of pH resistance. These included EGFP, monomeric EGFP (mEGFP), monomeric Clover3 (mClover3), monomeric NeoGreen (mNeoGreen), Gamillus, and monomeric GreenLantern (mGreenLantern). Under nutrient-rich conditions, basal CMA activity was relatively low, with all green fluorescent proteins evenly distributed in the cytoplasm. Notably, compared to other green fluorescent proteins, the KFERQ motif fused with the pH-resistant Gamillus variant (KFERQ-Gamillus) showed a pronounced accumulation of green fluorescent puncta upon serum deprivation (Figure 1A,B). These results indicate that KFERQ-Gamillus is a viable pH-resistant reporter for monitoring CMA activity in living cells.

### 2.2. KFERQ-Gamillus Is a CMA-Specific Fluorescent Reporter

**Figure 2:**
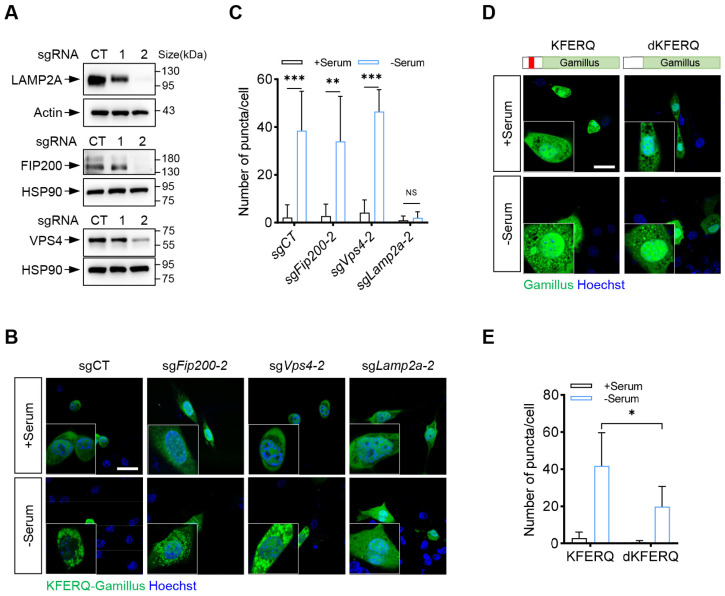
KFERQ-Gamillus is a CMA-specific fluorescent reporter. (**A**) Western blot analysis was used to detect the knockout efficiency in NIH-3T3 cells stably expressing the control or two independent sgRNAs targeting *Fip200*, *Vps4,* and *Lamp2a*. (**B**) and (**C**) NIH-3T3 KFERQ-Gamillus cells stably expressing the control or sgRNAs targeting *Fip200*, *Vps4,* and *Lamp2a*, as indicated, were cultured in medium with (+Serum) or without (−Serum) serum for 24 h. Shown are representative cell images (**B**) and average number of puncta per cell (**C**) (*n* =12 for each of three independent experiments). Scale bar, 10 μm. (**D**,**E**) NIH-3T3 cells stably expressing KFERQ-Gamillus or dKFERQ-Gamillus were cultured in medium with (+Serum) or without (-Serum) serum for 24 h. Shown are representative cell images (**D**) and average number of puncta per cell (**E**) (*n* = 8 for each of three independent experiments). Scale bar, 10 μm. Data are shown as mean ± SD. NS, not significant; * *p* < 0.05, ** *p* < 0.01, and *** *p* < 0.001; unpaired Student’s *t*-test.

Next, we assessed whether this reporter is specific to the CMA pathway. First, we constructed stable cell lines lacking a key macroautophagy regulator (Fip200), an ATPase essential for microautophagy (Vps4), or the rate-limiting receptor crucial for CMA (Lamp2a) (Figure 2A). Compared to the control, both Fip200 and Vps4 knockout cells showed no significant changes; however, the depletion of Lamp2a significantly inhibited the dot-like accumulation of KFERQ-Gamillus in NIH-3T3 cells cultured under serum-deprived conditions (Figure 2B,C). Additionally, in Lamp2a KO cells, the fluorescence of KFERQ-Gamillus predominantly remained as a diffuse cytosolic pattern, even after serum removal (Figure 2B,C). The absence of KFERQ-Gamillus puncta in Lamp2a KO cells further supports that this reporter relies on functional CMA to reach lysosomes. These findings collectively indicate that KFERQ-Gamillus serves as a specific reporter for CMA activity.

CMA substrates contain a KFERQ pentapeptide motif, which is recognized by the cytosolic chaperone HSC70. To further confirm whether the KFERQ pentapeptide motif is essential for the delivery and subsequent degradation of KFERQ-Gamillus in the lysosome, we deleted the five KFERQ amino acids from KFERQ-Gamillus, creating dKFERQ-Gamillus. We then analyzed the cells using immunofluorescence under serum-rich and serum-deprived conditions. As expected, the removal of the KFERQ motif significantly decreased CMA activity under serum-deprived conditions (Figure 2D,E). Together, these results suggest that KFERQ-Gamillus is a robust fluorescent reporter for monitoring CMA activity.

### 2.3. Detection of CMA Activity with the KFERQ-Gamillus Fluorescent Probe Using Image-Based Flow Cytometry

**Figure 3:**
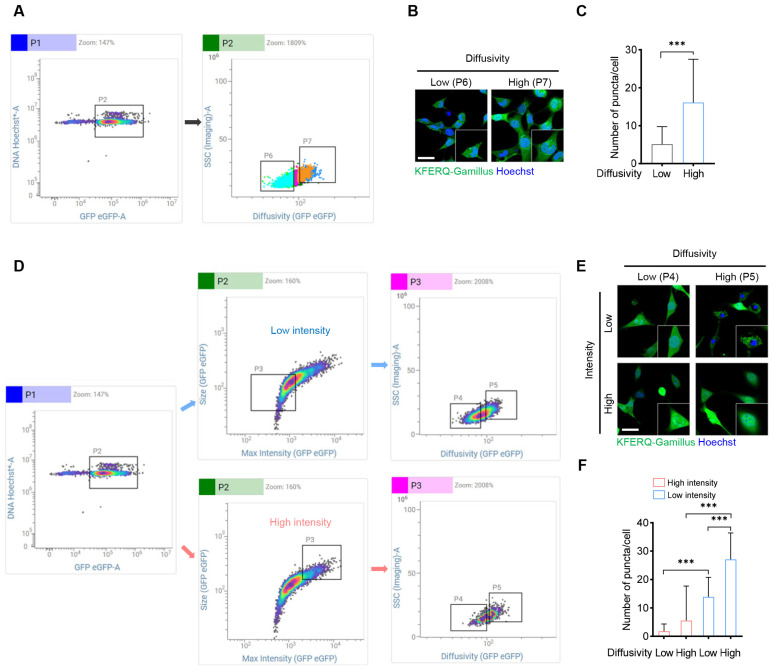
KFERQ-Gamillus is suitable for analyzing CMA activity in an image-based cell sorter. (**A**–**C**) NIH-3T3 cells stably expressing KFERQ-Gamillus were incubated with Hoechst for nuclear staining and subsequently analyzed using image-based flow cytometry. The selected P2 cell population was sorted based on KFERQ-Gamillus diffusivity, with P6 and P7 representing cells with low and high diffusivity, respectively. The sorted P6 and P7 cells were cultured for several hours until fully attached, followed by immunofluorescence (IF) analysis; representative images of the cells are shown in panel (**B**), and the average number of puncta per cell is quantified in panel (**C**) (*n* = 30 for each of three independent experiments). Scale bar: 10 μm. (**D**–**F**) The selected P2 cell population (same as in A) was first sorted based on intensity of KFERQ-Gamillus, and the selected P3 cell population was subsequently sorted based on KFERQ-Gamillus diffusivity, with P4 and P5 representing cells with low and high diffusivity, respectively. The sorted P4 and P5 cells, with low or high intensity, were cultured for several hours until fully attached, followed by immunofluorescence analysis; representative images of the cells are shown in panel (**E**), and the average number of puncta per cell is quantified in panel (**F**) (*n* = 25 for each of three independent experiments). Scale bar: 10 μm. Data are shown as mean ± SD, with *** *p* < 0.001, as determined by an unpaired Student’s *t*-test.

Fluorescence-activated cell sorting (FACS) offers a viable alternative for tracking CMA activity using fluorescent reporters, especially in cell types that are challenging to analyze via fluorescence-microscopy-based morphometrics. However, the rapid and selective isolation of single cells with distinct spatial and morphological features remains difficult. Recently, a high-speed fluorescence-image-enabled cell-sorting approach was developed [32]. To assess whether KFERQ-Gamillus could serve as an effective reporter for CMA activity in an image-based flow cytometry setting, we utilized the dispersion feature to sort cell populations based on the distribution of green fluorescent spots (Figure 3A). Our findings indicated that cells with high diffusivity exhibited increased CMA activity, marked by more green puncta, as confirmed by confocal immunofluorescence (Figure 3B,C). However, this feature alone did not fully distinguish between high and low CMA activity populations, as some cells with high CMA activity were still observed in low-diffusivity populations under the confocal microscope (Figure 3B).

Considering that average fluorescence intensity decreases with elevated CMA activity [11], we then combined both intensity and diffusivity indices for more refined cell sorting. First, we categorized cells into low and high fluorescence intensity groups and further sorted each by diffusivity (Figure 3D). This two-step approach significantly improved sorting accuracy, as confirmed by immunofluorescence: cells with high diffusivity and low average fluorescence intensity displayed high CMA activity, while cells with low diffusivity and high fluorescence intensity exhibited low CMA activity populations (Figure 3E,F). Together, these results validate KFERQ-Gamillus as an effective reporter for image-enabled cell sorting, making it a promising tool for high-throughput screening applications, including genome-wide screening assays for immune cells.

### 2.4. KFERQ-Gamillus-Halo Serves as an Effective Reporter for Assessing CMA Flux in Living Cells

**Figure 4:**
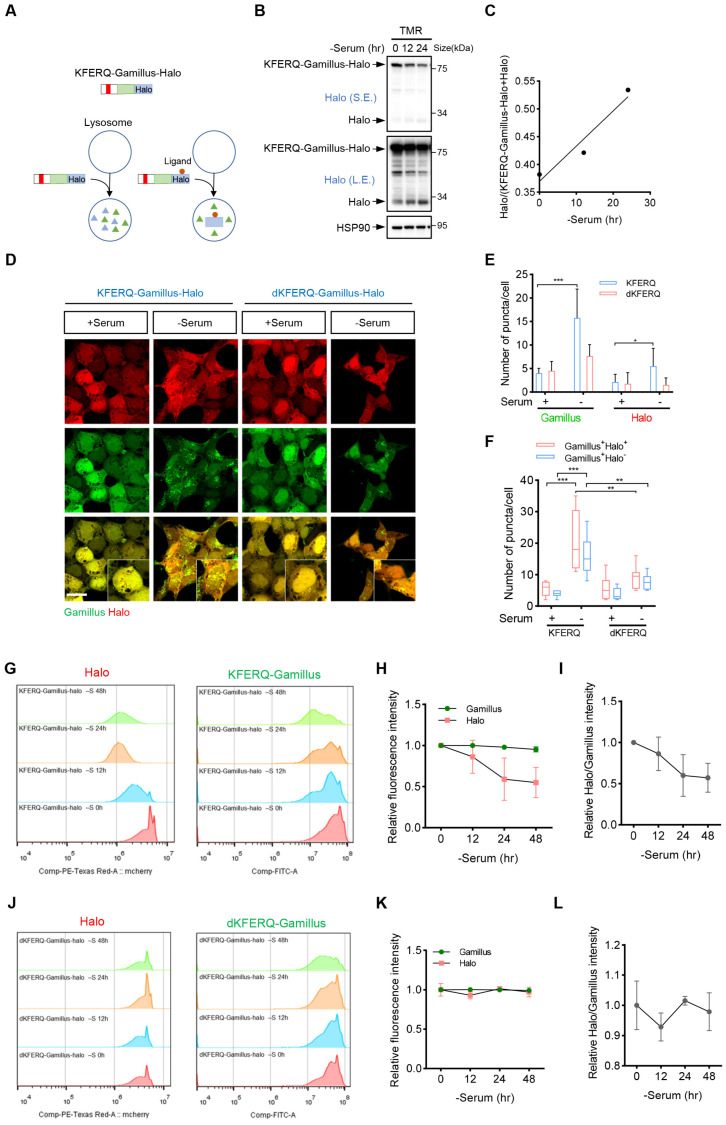
Evaluation of CMA flux with KFERQ-Gamillus-Halo. (A) Schematic representation of the fate of ligand-free and ligand-bound KFERQ-Gamillus-Halo. (**B**,**C**) HEK293 cells stably expressing KFERQ-Gamillus-Halo were cultured in serum-deprived medium for the indicated times. The cells were then pulse-labeled for 20 min with 10 nM of Tetramethylrhodamine (TMR)-conjugated ligand, and cell lysates were analyzed by Western blot (**B**). The Halo^TMR^ band intensity was quantified by normalizing it to the combined intensity of the KFERQ-Gamillus-Halo^TMR^ and Halo^TMR^ bands (**C**). S.E., short exposure; L.E., long exposure. (**D**–**F**) HEK293 cells stably expressing KFERQ-Gamillus-Halo or dKFERQ-Gamillus-Halo were cultured with (+Serum) or without (−Serum) for 24 h. Following this, cells were pulse-labeled for 20 min with 10 nM of TMR-conjugated Halo ligand and then analyzed by immunofluorescence. Panel (**D**) shows representative images of cells, while panel (**E**) presents the average number of puncta per cell for Gamillus and Halo^TMR^ (*n* = 8 for each of three independent experiments). Panel (**F**) quantifies Gamillus^+^Halo^TMR+^ and Gamillus^+^Halo^TMR-^ puncta, representing substrates associated with lysosomes (*n* =8 for each of three independent experiments). Scale bar = 10 μm. (**G**–**L**) HEK293 cells stably expressing KFERQ-Gamillus-Halo (**G**–**I**) or dKFERQ-Gamillus-Halo (**J**–**L**) were cultured without serum (-S) for the indicated times; the cells were then pulse-labeled for 20 min with 10 nM of TMR-conjugated ligand and further analyzed by flow cytometry. Shown are mean Gamillus fluorescence intensity (**G**,**J**), the relative fluorescence intensity (**H**,**K**), and the relative Halo^TMR^/Gamillus fluorescence intensity (**I**,**L**). Data are shown as mean ± SD; * *p* < 0.05, ** *p* < 0.01, and *** *p* < 0.001; unpaired Student’s *t*-test.

Monitoring autophagic flux is crucial for most autophagy studies; however, to our knowledge, there are currently no fluorescent-protein-based reporters available to assess CMA flux in living cells. Previously, Halo-Tag-based reporters have been used to monitor autophagy activity or flux. For example, a Halo-Tag-based LC3B reporter assay was developed to measure macroautophagic flux in mammalian cells [30]. Additionally, GAPDH, a known CMA substrate, fused to Halo-Tag has been used to effectively monitor CMA activity through immunofluorescence in single neurons [31]. Since Halo-Tag alone is prone to lysosomal degradation but becomes resistant upon ligand binding, the presence of free Halo ligands, detected by immunoblotting, can serve as an indicator of autophagic flux (Figure 4A). We stably expressed the GAPDH-Halo reporter in HeLa cells and exposed these cells to Tetramethylrhodamine (TMR)-conjugated Halo ligand, observing that serum starvation significantly increased CMA activity (Figure S1A,B), consistent with previous findings [31]. To further evaluate whether CMA flux can be monitored via immunoblotting, these cells were incubated with Halo ligands, which were subsequently washed out. After 24 h of serum starvation, a starvation-induced decrease in both labeled and unlabeled GAPDH-Halo was observed via immunoblotting, with free Halo detected exclusively for GAPDH-Halo^TMR^ (Figure S1C). Additionally, a small fraction of free Halo^TMR^ was detected in serum-starved NIH-3T3 cells expressing GAPDH-Halo but not in Lamp2a knockout cells, suggesting that the generation of free Halo^TMR^ was CMA-dependent (Figure S1D).

Currently, isolating lysosomes and performing in vitro reconstitution is the standard method available for tracking the degradation steps in CMA flux processes, including substrate binding, lysosomal uptake, and degradation. However, this approach requires multiple steps to purify both lysosomes and substrates, cannot be conducted directly in living cells, and is generally inefficient. To overcome these limitations, we constructed a KFERQ-Gamillus-Halo reporter and then tested whether it could be used to monitor CMA flux in mammalian cells. We stably expressed this reporter in HEK293 or NIH-3T3 cells and exposed these cells to TMR-conjugated ligands. A starvation-induced decrease in labeled and unlabeled KFERQ-Gamillus-Halo was observed via immunoblotting, with increased free Halo^TMR^ detected (Figure 4B,C and Figure S2A,B).

Next, we assessed CMA activity in cells stably expressing KFERQ-Gamillus-Halo and dKFERQ-Gamillus-Halo using the immunofluorescence of Gamillus or Halo ligands under both control and serum-deprived conditions. As Halo^TMR^ is relatively more stable than KFERQ-Gamillus in lysosomes after lysosomal digestion, puncta in the Halo^TMR^ channel reflect accumulated substrates, making it particularly suitable for detecting cells with low basal CMA activity. As anticipated, serum deprivation led to an increase in dot-like accumulations of both Gamillus and Halo^TMR^ puncta, while the deletion of the KFERQ sequence significantly reduced these accumulations (Figure 4D,E and Figure S2C,D). Additionally, the number of colocalized Halo^TMR^-positive and Gamillus-positive puncta increased, suggesting that KFERQ-Gamillus-Halo translocates to lysosomes, as visualized using the Halo ligand. In contrast, the deletion of the KFERQ sequence significantly reduced this colocalization (Figure 4F and Figure S2E).

We further assessed CMA activity using flow cytometry. Serum deprivation gradually decreased the Halo^TMR^ fluorescence intensity in cells stably expressing the KFERQ-Gamillus-Halo reporter, whereas deletion of the KFERQ sequence partially blocked this reduction (Figure 4G–L). However, the decrease in KFERQ-Gamillus intensity was relatively mild compared to Halo^TMR^ (Figure 4G–L and Figure S2F–H). Previously, the GFP-LC3-RFP-LC3ΔG fluorescent probe was used to estimate macroautophagy flux based on the GFP/RFP signal ratio, with RFP-LC3ΔG serving as an internal control [29]. Given that Halo^TMR^-positive puncta specifically indicate substrates that associate with lysosomes only after the addition of the Halo ligand, we calculated the Halo/Gamillus signal ratio and observed that this value accurately reflects changes in CMA flux (Figure 4I,L). Furthermore, treatment with the CMA activator CA77 also slightly reduced the Gamillus fluorescence intensity in NIH-3T3 cells, and serum deprivation further exacerbated this reduction (Figure S2I,J). Taken together, these results indicate that KFERQ-Gamillus-Halo is an effective reporter for monitoring CMA flux.

## 3. Discussion

Developing robust and dynamic markers to track chaperone-mediated autophagy is crucial for elucidating the complexities of selective autophagy and understanding its roles in cellular homeostasis, aging, and disease. In this study, we developed KFERQ-Gamillus, a promising pH-resistant marker for CMA activity, by screening various KFERQ-fused green fluorescent proteins. This reporter specifically responds to known CMA activators, such as serum deprivation, and relies on LAMP2A and the KFERQ motif for lysosomal localization and degradation, highlighting its specificity for the CMA pathway. We further demonstrated the versatility of KFERQ-Gamillus by adapting it for image-based flow cytometry to sort cell populations based on CMA activity, using fluorescence intensity and diffusivity metrics. Additionally, we developed a dual-reporter system, KFERQ-Gamillus-Halo, which can distinguish between protein synthesis and degradation, and enables the measurement of intracellular CMA flux via immunoblotting and flow cytometry, without the need for lysosomal inhibitors. In summary, our findings suggest that KFERQ-Gamillus and KFERQ-Gamillus-Halo are valuable tools for advancing our understanding of CMA’s physiological and pathological roles, with potential applications in identifying CMA modulators and developing therapeutic strategies targeting CMA for disease treatment.

The KFERQ-Gamillus-Halo reporter developed in this study offers several key advantages over existing CMA activity detection reporters and assays (Table 1). Its ability to monitor CMA flux in living cells using a pulse–chase approach is particularly advantageous. This design minimizes background noise, as unlabeled reporters do not accumulate in lysosomes. Free Halo detection is LAMP2A- and KFERQ-motif-dependent, occurring only after the reporter is labeled, transported to, and processed in lysosomes. Additionally, the stabilization of Halo upon ligand binding in lysosomes protects it from proteolysis, enabling the detection of CMA flux in cells with low basal CMA activity. The probe does not require light activation and supports flexible color selection via the Halo ligand, enabling the simultaneous detection of multiple indicators. For example, differently colored Halo probes can be added or removed at various time points to dynamically track changes in CMA activity under different conditions. Furthermore, Gamillus itself is resistant to lysosomal pH fluctuations, preventing pH-related interference with CMA activity. Another key advantage is the elimination of lysosome inhibitor controls, which could interfere with cellular proteome homeostasis. Reporter processing can be easily detected using commonly available laboratory techniques, such as immunoblotting, immunofluorescence, or FACS. Finally, by replacing Halo’s partner protein, users can easily adapt the assay to monitor the CMA-mediated clearance of specific substrate proteins of interest.

We further tested the KFERQ-Gamillus probe to differentiate CMA activity levels using an image-based flow sorting system. By utilizing both fluorescence intensity and dispersion properties in the cytometer, we accurately distinguished cells with high and low CMA activity. This feature is particularly useful for detecting CMA activity in suspension cells, such as immune cells, without the need for traditional immunofluorescence. Moreover, similar to the GFP-LC3-RFP-LC3△G probe, the stable fluorescence intensity of Gamillus allows for the indirect measurement of CMA flux by observing changes in the Halo/Gamillus fluorescence ratio. Thus, our reporter system integrates features from existing CMA activity detection methods while simplifying CMA flux detection, reducing limitations, and significantly expanding potential applications. This new tool provides a valuable resource for high-throughput drug screening and the discovery of novel CMA regulatory factors.

Despite these advances, several challenges remain to be addressed. One limitation is the reliance on exogenous expression, and the cost of using Halo ligands is still considerable. Additionally, accurately quantifying the absolute rate of reporter incorporation into lysosomes is challenging due to the gradual degradation of even ligand-bound Halo. Future research could investigate the application of KFERQ-Gamillus-Halo across diverse cell types, including primary cells and disease-relevant models, to validate its versatility and broad applicability in settings such as high-throughput drug discovery and genome-wide screening. A direct comparison of the performance of this system with existing CMA detection methods is also essential to assess its relative efficacy. Furthermore, ensuring that the overexpression of this reporter does not interfere with normal cellular processes is critical, which can be evaluated using RNA sequencing or proteomics analysis. Expanding this system to include multiplexed reporters could enable the simultaneous tracking of CMA alongside other autophagic or cellular processes, offering deeper insights into the complex interplay between CMA and related pathways. Finally, as Halo-based reporters have been successfully applied in in vivo models [33,34,35,36], adapting this probe for detecting CMA flux in in vivo contexts would significantly advance our understanding of dynamic CMA activity across various tissues.

## 4. Materials and Methods

### 4.1. Cell Lines and Culture

NIH-3T3, HeLa, HEK293, and HEK-293T cell lines were obtained from the American Type Culture Collection (ATCC, Manassas, VA, USA). The cells were maintained in Dulbecco’s Modified Eagle Medium (DMEM; Thermo Fisher Scientific, Fair Lawn, NJ, USA, 12100046,), supplemented with 10% fetal bovine serum (FBS; Braserum, Hangzhou, China, BX0500CA) and 1% Penicillin/Streptomycin (Thermo Fisher Scientific, Fair Lawn, NJ, USA, 15140122), and cultured in a humidified 5% CO_2_ incubator at 37 °C.

### 4.2. Plasmid Construction

To construct KFERQ-fused green fluorescent protein expression plasmids, a DNA fragment encoding a 21-amino acid region of RNase A (MKETAAAKFERQHMDSSTSAA), which includes the KFERQ motif, was synthesized (5′-atgaaggaaactgcagcagccaagtttgagcggcagcacatggactccagcacttccgctgcg-3′) [11]. This fragment was fused to sequences encoding various green fluorescent proteins, including enhanced green fluorescent protein (EGFP), monomeric enhanced GFP with an A206K mutation (mEGFP), monomeric Clover3 (mClover3), monomeric NeoGreen (mNeoGreen), Gamillus, and monomeric GreenLantern (mGreenLantern). The fusion constructs were synthesized by Genescript (Nanjing, China) and cloned into the pCDH-EF1-FHC vector (Addgene, Watertown, MA, USA, #64874) using EcoRI and BamHI restriction sites. To construct the dKFERQ-Gamillus plasmid, DNA fragments encoding dKFERQ and Gamillus were amplified using Vazyme polymerase (P515) by polymerase chain reaction (PCR) from the KFERQ-Gamillus plasmid with the following primers: dKFERQ-Forward: 5′-GCTAGCGAATTCGCCACCATGAAGGAAACTGCAGCAGCC-3′; dKFERQ-Reverse:5′-ATGCCTCCTCGCCCTTGCTCACCATCGCAGCGGAAGTGCTGGAGTCCATG-3′; Gamillus-Forward:5′-CATGGACTCCAGCACTTCCGCTGCGATGGTGAGCAAGGGCGAGGAGGCAT-3′; Gamillus-Reverse: 5′-GAAAGCCAGTACCGATTTCTGCCATCTTGTACAGCTCGTCCATGCCGTGC-3′. The purified DNA fragments of dKFERQ and Gamillus were mixed at a 1:1 ratio and amplified by PCR using dKFERQ-forward and Gamillus-reverse primers. The resulting sequence was inserted into the pCDH-EF1-FHC vector at the EcoRI and BamHI sites. For the KFERQ-Gamillus-Halo plasmid, DNA fragments encoding dKFERQ, Gamillus, and Halo were amplified by PCR. The Halo tag was amplified from the pYT-Halo-C plasmid (Addgene #178137) using the following primers: Halo-Forward: 5′-GCACGGCATGGACGAGCTGTACAAGATGGCAGAAATCGGTACTGGCTTTC-3′; Halo-Reverse:5′-GGCCGCGATCCGCCGGAAATCTCGAGCGTCGACAGC-3′. Purified DNA fragments of dKFERQ, Gamillus, and Halo were mixed at a 1:1:1 ratio and cloned into the pCDH-EF1-FHC vector using homologous recombination with the ClonExpress Ultra One Step Cloning Kit V2 (Vazyme, Nanjing, China, C116-01).

To construct the GAPDH-Halo plasmid, DNA fragments encoding GAPDH and Halo were amplified by PCR from HEK293 cDNA and the pYT-Halo-C plasmid, respectively, using these primers: GAPDH-Forward: 5′-GCTAGCGAATTCGCCACCATGGTTTACATGTTCCAATATGATT-3′; GAPDH-Reverse:5′-CGAATGGAAAGCCAGTACCGATTTCTGCCATCTCCTTGGAGGCCATGTAGGCCATG-3′; Halo-Forward:5′-CATGGCCTACATGGCCTCCAAGGAGATGGCAGAAATCGGTACTGGCTTTCCATTCG-3′; Halo-Reverse: 5′-GGCCGCGGATCCACCGGAAATCTCCAGAGTAGACAGC-3′. The purified DNA fragments of GAPDH and Halo were mixed at a 1:1 ratio and amplified by PCR with GAPDH-forward and Halo-reverse primers. The resulting sequence was inserted into the pCDH-EF1-FHC vector at the EcoRI and BamHI sites. To construct the KFERQ-Gamillus-Halo plasmid, DNA fragments encoding KFERQ-Gamillus and Halo were amplified by PCR from the KFERQ-Gamillus and pYT-Halo-C plasmids using these primers: KFERQ-Gamillus-Forward: 5′-GCTAGCGAATTCGCCACCATGAAGGAAACTGCAGCAGCCAAGT-3′; KFERQ-Gamillus-Reverse:5′-GAAAGCCAGTACCGATTTCTGCCATCTTGTACAGCTCGTCCATGCCGTGC-3′. The purified fragments of KFERQ-Gamillus and Halo were mixed at a 1:1 ratio and cloned into the pCDH-EF1-FHC vector using homologous recombination with the ClonExpress Ultra One Step Cloning Kit V2 (Vazyme, Nanjing, China, C116-01).

### 4.3. Lentiviral Packaging and Stable Cell Line Construction

sgRNAs were first cloned into the lentiCRISPRv2 vector (Addgene, Watertown, MA, USA, #52961); sgRNA sequences are listed in Table S1. To construct Fip200, Vps4, and Lamp2a KO or KFERQ-fused green fluorescent protein expression cell lines, the virus was generated by transient transfection of lentiCRISPRv2 or overexpression vectors, together with the packaging plasmids and the constructs that express the vesicular stomatitis virus glycoprotein by using polyethyleneimine (PEI, Polysciences, Warrington, PA, USA) reagent. After 24 h, virus particles in the medium were collected, filtered, and added to the cells. Cells were cultured in the presence of antibiotics for 7 days to generate stable cell lines.

### 4.4. Antibodies and Reagents

The primary antibodies used in this study were as follows: mouse monoclonal anti-Halo (Promega, Madison, WI, USA, G9211, 1:1000), anti-LAMP2A (Abcam, Cambridge, UK, ab125068, 1:1000), anti-HSP90 (Origene, Rockville, MD, USA, TA500494, 1:1000), anti-β-actin (Santa Cruz, Dallas, TX, USA, sc-69879, 1:1000); rabbit polyclonal anti-VPS4 (Sigma, St. Louis, MA, USA, SAB4200025, 1:1000); and anti-RB1CC1 (Proteintech, Wuhan, China, 17250-1-AP, 1:1000). Secondary antibodies included HRP-conjugated goat polyclonal anti-rabbit IgG (Cell Signaling Technology, Danver, TX, USA, 7074S, 1:2000) and HRP-conjugated goat polyclonal anti-mouse IgG (Cell Signaling Technology, Danver, TX, USA, 7076S, 1:2000). All antibodies were diluted in 5% BSA (Bovine Serum Albumin) prepared in PBST (phosphate-buffered saline with Tween 20). Halo Tag Tetramethylrhodamine (TMR) ligand was purchased from Promega, Madison, WI, USA (G8252).

### 4.5. Western Blotting

Cells were incubated in DMEM supplemented with 10 nM of TMR-conjugated Halo ligand for 10 min at 37 °C. Following incubation, cells were washed twice with phosphate-buffered saline (PBS) and collected for protein extraction. Protein concentrations were quantified using the BCA Protein Assay Kit (Thermo Fisher Scientific, Fair Lawn, NJ, USA, A55865) according to the manufacturer’s instructions. For each sample, 20 μg of total protein was separated by 8%~12% SDS-PAGE and subsequently transferred to a PVDF membrane (Sigma, St. Louis, MA, USA) using the Bio-Rad Transfer System. Membranes were blocked with 5% non-fat milk in PBST and incubated with primary and secondary antibodies specific to target proteins. Signals were visualized using the Super ECL Detection Reagent (YEASEN, Shanghai, China, 36208ES60) and imaged on an automated system (Tanon, Shanghai, China, 5200). Band intensities were analyzed to calculate the relative ratios of the indicated proteins to loading controls, such as actin or Hsp90. These ratios were subsequently normalized to the corresponding values in the control sample. Western blot quantification was performed using the ImageJ software (Version 1.54b, National Institutes of Health, Bethesda, MD, USA).

### 4.6. Immunofluorescence Imaging

To assess CMA activity, cells stably expressing KFERQ-fused green fluorescent protein (GFP) constructs or GAPHD-Halo were seeded onto glass-bottom confocal dishes (BIOFIL, Guangzhou, China, BDD011035) and cultured in either complete growth medium or serum-free medium to induce CMA. Twenty minutes before imaging, cells were incubated with 10 nM Halo-Tag TMR Ligand and Hoechst 33342 (Thermo Fisher Scientific, Fair Lawn, NJ, USA, H1399). Imaging was acquired using a Zeiss LSM 880 confocal microscope (Carl Zeiss, Oberkochen, Germany). CMA activity was assessed by analyzing changes in the cellular distribution pattern of the reporter, with fluorescent puncta per cell quantified in ImageJ; the number of puncta per cell served as an indicator of CMA activity.

### 4.7. Flow Cytometry Analysis

Cells were detached from culture plates using trypsin-EDTA solution (Thermo Fisher Scientific, Fair Lawn, NJ, USA, 25200072), neutralized with DMEM, and centrifuged at 300× *g* for 3 min at 4 °C. The cell pellet was resuspended in phenol red-free DMEM (Thermo Fisher Scientific, Fair Lawn, NJ, USA, 21063029) for flow cytometry. Flow cytometry analysis was performed using a Cytek Aurora cell analyzer (Fremont, CA, USA). Data acquisition and analysis were conducted using the FlowJo software (Version 1.54b, National Institutes of Health, Bethesda, MD, USA), and image-based cell sorting was performed with the BD FACS Discover™ S8 system (Franklin Lakes, NJ, USA).

### 4.8. Statistical Analysis

All numerical results are presented as mean ± standard deviation (SD) and represent data from a minimum of three independent experiments unless otherwise stated. Unpaired two-tailed Student’s *t*-tests or ANOVA were used to evaluate statistical significance. *p*-values were calculated using the GraphPad Prism 9 software (Boston, MA, USA), and values < 0.05 were considered statistically significant.

## 5. Conclusions

In this study, we developed and validated KFERQ-Gamillus and KFERQ-Gamillus-Halo as effective and versatile tools for monitoring the activity and flux of chaperone-mediated autophagy in living cells, addressing critical challenges in the field. The pH-resistant KFERQ-Gamillus probe exhibited high specificity for the CMA pathway, enabling real-time tracking of lysosomal localization and degradation under conditions that activate CMA, such as serum starvation. Its strong performance in image-based flow cytometry further underscores its suitability for high-throughput screening and the identification of cell populations with varying levels of CMA activity. We also developed the dual-reporter system KFERQ-Gamillus-Halo by integrating KFERQ-Gamillus with the Halo-tag system. This system facilitates the quantitative assessment of CMA flux through immunoblotting and rapid CMA activity analysis via flow cytometry, without requiring lysosomal inhibitors. Notably, it distinguishes between protein synthesis and degradation, offering a comprehensive and dynamic perspective on CMA activity in living cells. These tools represent a meaningful contribution to CMA research, providing real-time, accurate, and quantitative tools for detecting CMA activity and flux. The KFERQ-Gamillus and KFERQ-Gamillus-Halo reporters hold great potential for applications extending beyond basic research, including high-throughput screening; drug discovery; and the exploration of diseases associated with CMA dysregulation, such as aging, cancer, and neurodegenerative disorders. By addressing the limitations of existing detection methods, these tools will help advance our understanding of the biological function of CMA and explore its potential in disease treatment.

## Figures and Tables

**Table 1:** Comparative analysis of CMA detection tools.

Tool	Advantages	Disadvantages
PA-KFERQ-mCherry	High specificity for CMA activity, low background, and can detect CMA activity in living cells.	Requires photoexcitation and is prone to photobleaching.
	Specifically monitors protein degradation and is not interfered with by new protein synthesis.	Unsuitable for detecting low CMA activity, high-throughput screening, suspension cells, and long-term observations.
	No need to add fluorescent ligands.	Cannot directly detect CMA flux.
KFERQ-Dendra	Detects CMA activity in vivo.	Unsuitable for detecting low CMA activity.
	Suitable for time-lapse experiments.	Cannot directly detect CMA flux.
GAPDH-Halo	Highly flexible and can be combined with various fluorescent probes.	GAPDH is a broadly expressed protein that does not fully reflect CMA specificity.
	Detects flux via immunoblotting.	May be influenced by protein aggregation or other signaling pathways that regulate GAPDH expression.
KFERQ-AMC	Detects CMA activity in cells and tissues.	Requires the addition of lysosomal inhibitors to measure CMA flux.
	Easy detection using a microplate reader.	Requires lysosome extraction, which takes too long and is unsuitable for real-time and living cell detection.
KFERQ-Gamillus-Halo	pH-resistant and highly flexible; can be combined with various fluorescent probes.	Further in vivo verification of CMA activity and flux measurements is needed.
	Detects low intracellular CMA flux via immunoblotting or flow cytometry.	Requires adding Halo ligands to detect CMA flux, which can result in background signal interference.
	Suitable for high-throughput screening or sorting of cells with different CMA activity.	
	Capable of distinguishing between protein synthesis and degradation.	

## Data Availability

The experimental data presented in the study are included in the article/Supplementary Materials; further inquiries can be directed to the corresponding authors.

## Supplementary Materials

The following supporting information can be downloaded at https://www.mdpi.com/article/10.3390/ijms26010017/s1.
