# Supplementary material for: Tracking Chaperone-Mediated Autophagy Flux with a pH-Resistant Fluorescent Reporter
Source: Int J Mol Sci. 2024 Dec 24;26(1):17. doi: 10.3390/ijms26010017 (PMC11719817; doi:10.3390/ijms26010017)
Supplement: Supplementary file 1 [file ijms-26-00017-s001.zip › ijms-3349230-supplementary.pdf]

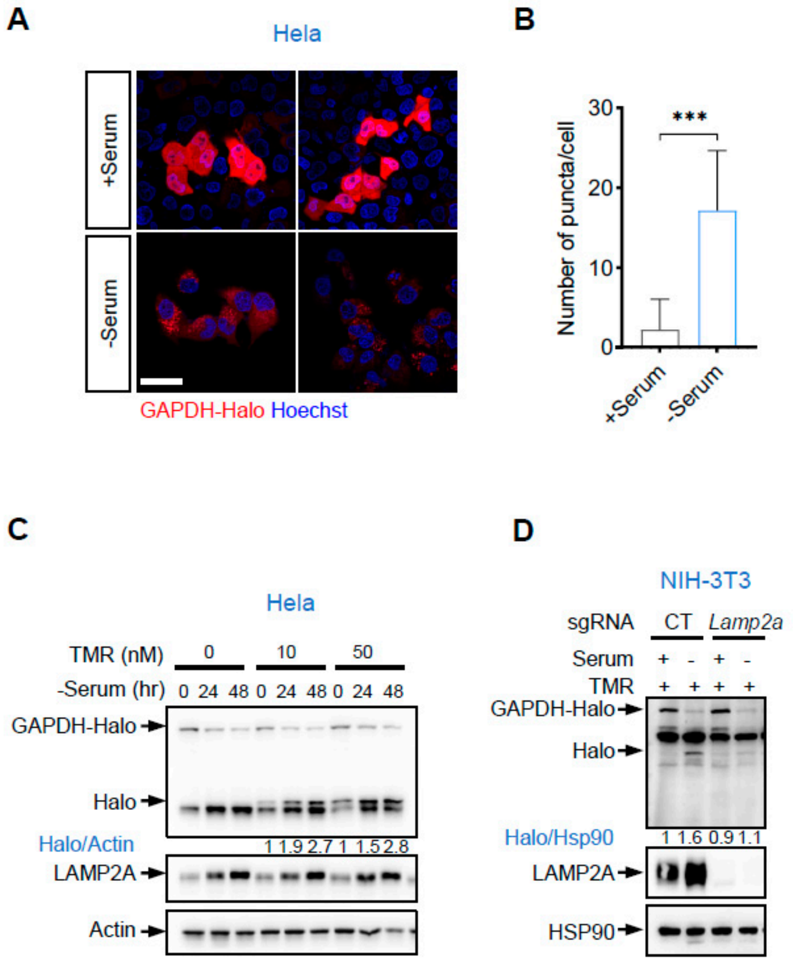

Fig. S1. Evaluation of CMA flux with GAPDH-Halo.

A and **B**, Hela cells stably expressing GAPDH-Halo were cultured with (+Serum) or without serum (-Serum) for 24 hours. The cells were then pulse-labeled for 20 minutes with 10 nM of tetramethylrhodamine (TMR)-conjugated ligand, and cell were analyzed by IF, shown are representative cell images (**A**) and average number of puncta per cell (**B**, n =16 for each of three independent experiments). Scale bar, 10 μm.

C, Hela cells stably expressing GAPDH-Halo were cultured in serum-deprived medium for the indicated times. The cells were then pulse-labeled with different concentration of TMR-conjugated ligand, and cell lysates were analyzed by Western blot.

D, NIH-3T3 cells stably expressing control or *Lamp2a* sgRNA were transfected with GAPDH-Halo plasmids and were further cultured with or without serum for 24 hours. The cells were then pulse-labeled TMR-conjugated ligand, and cell lysates were analyzed by Western blot.

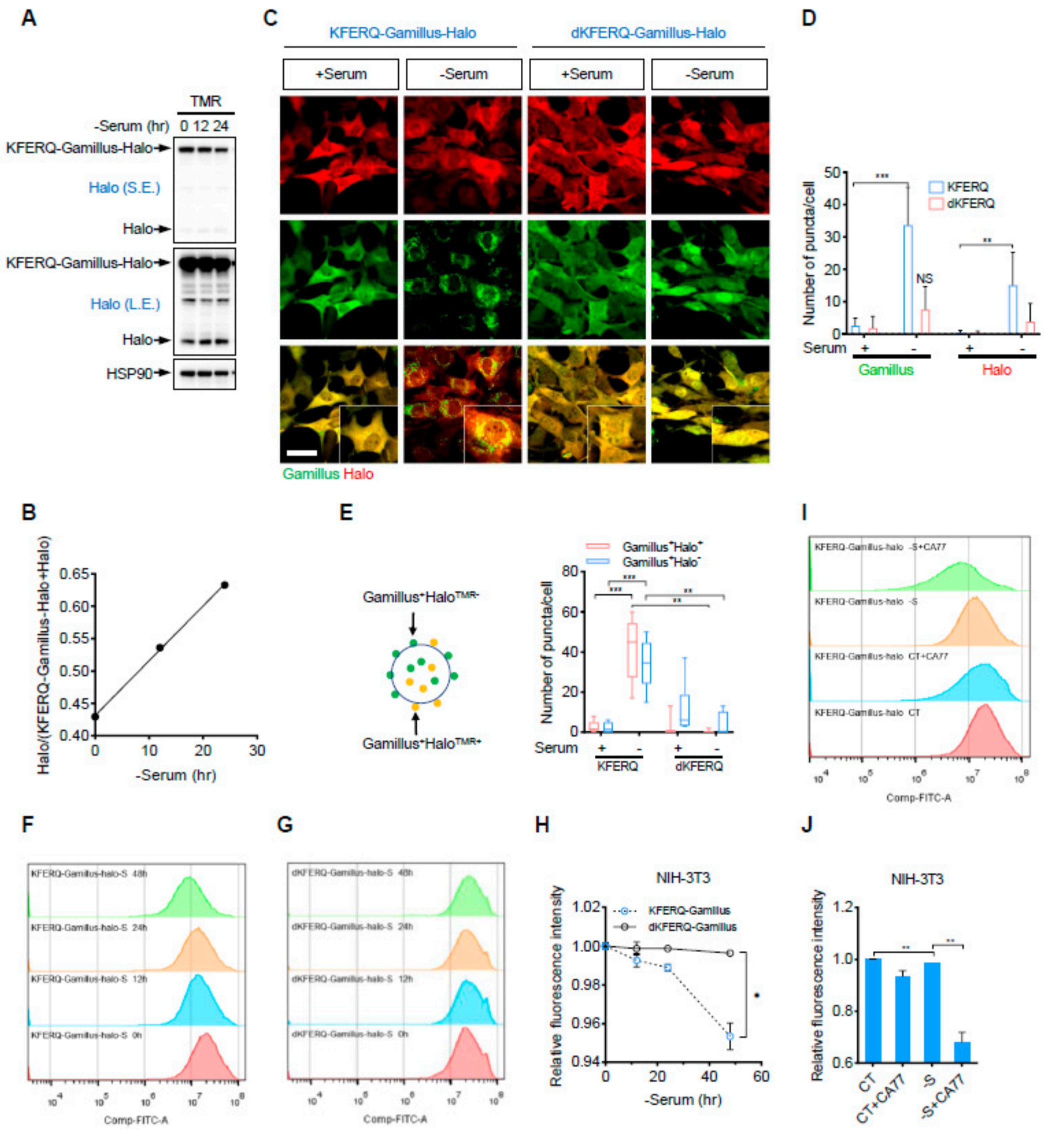

Fig. S2. Evaluation of CMA flux with KFERQ-Gamillus-Halo in NIH-3T3 cells.

A and **B**, NIH-3T3 cells stably expressing KFERQ-Gamillus-Halo were cultured in serum-deprived medium for the indicated times. The cells were then pulse-labeled for 20 minutes with 10 nM of TMR-conjugated ligand, and cell lysates were analyzed by Western blot (**A**). The Halo^{TMR} band intensity was quantified by normalizing it to the combined intensity of the KFERQ-Gamillus-Halo^{TMR} and Halo^{TMR} bands (**B**). S.E, short exposure; L.E., long exposure.

C to **E**, NIH-3T3 cells stably expressing KFERQ-Gamillus-Halo or dKFERQ-Gamillus-Halo were cultured with (+Serum) or without (-Serum) for 24 hours. Following this, cells were pulse-labeled for 20 minutes with 10 nM TMR-conjugated Halo ligand and analyzed by immunofluorescence. Panel **C** shows representative images of cells, while Panel **D** presents the average number of puncta per cell for Gamillus and Halo^{TMR} (n =8 for each of three independent experiments). Panel **E** quantifies Gamillus^{+}Halo^{TMR+} and Gamillus^{+}Halo^{TMR-} puncta (n =8 for each of three independent experiments), representing two distinct pools of synthesized substrates associated with lysosomes. Gamillus^{+}Halo^{TMR+} puncta specifically

indicate substrates that became associated with lysosomes only after the addition of the Halo ligand TMR, while Gamillus^{+}Halo^{TMR-} puncta represent newly synthesized substrates that associated with lysosomes after the ligand was washed out. Scale bar = 10 µm.

F to **H**, NIH-3T3 cells stably expressing KFERQ-Gamillus-Halo (**F**) or dKFERQ-Gamillus-Halo (**G**) were cultured without serum (-S) for the indicated times, the cells were then analyzed by flow cytometry, shown are relative Gamillus fluorescence intensity (**H**).

I and **J**, NIH-3T3 cells stably expressing KFERQ-Gamillus-Halo cultured with (CT) or without (-S) serum were treated with or without CMA activator CA77 for 24 hours, the cells were then analyzed by flow cytometry, shown are mean Gamillus fluorescence intensity (**I**) and the relative mean Gamillus fluorescence intensity (**J**).

Data are mean + SD, NS, not significant, $*P < 0.05$, $**P < 0.01$, $***P < 0.001$; unpaired Student's *t* test.

Table S1. Sequences for sgRNA.

	**sgRNA sequence**	
**Name**	Forward	Reverse
sgLAMP2A-1	CACCGAGAGCTGCTCCCACCGCTAT	AAACATAGCGGTGGGAGCAGCTCTC
sgLAMP2A-2	CACCGATTGCTCATATCCAGTATGA	AAACTCATACTGGATATGAGCAATC
sgFip200-1	CACCGCGTTCAGTACCGGTGGGATC	AAACGATCCCACCGGTACTGAACGC
sgFip200-1	CACCGGTCAAATGTCAGCGTGGTTC	AAACGAACCACGCTGACATTTGACC
sgVps4a-1	CACCGACATCATTCCACCGTATGTT	AAACAACATACGGTGGAATGATGTC
sgVps4a-2	CACCGGTGACTCACATTTGATGGCG	AAACCGCCATCAAATGTGAGTCACC
sgVps4b-1	CACCGATGTCACCTGTAAAAAGATG	AAACCATCTTTTTACAGGTGACATC
sgVps4b-2	CACCGCAGCGCAAGAAGACAAGGCT	AAACAGCCTTGTCTTCTTGCGCTGC
